# Role of ceramide in diabetes mellitus: evidence and mechanisms

**DOI:** 10.1186/1476-511X-12-98

**Published:** 2013-07-08

**Authors:** Sehamuddin Galadari, Anees Rahman, Siraj Pallichankandy, Alaa Galadari, Faisal Thayyullathil

**Affiliations:** 1 Department of Biochemistry, Cell Signaling Laboratory, College of Medicine and Health Sciences, UAE University, P.O. Box 17666, Al Ain, Abu Dhabi, United Arab Emirates

**Keywords:** Diabetes, Sphingolipid, Ceramide, Insulin resistance, Pancreatic apoptosis

## Abstract

Diabetes mellitus is a metabolic disease with multiple complications that causes serious diseases over the years. The condition leads to severe economic consequences and is reaching pandemic level globally. Much research is being carried out to address this disease and its underlying molecular mechanism. This review focuses on the diverse role and mechanism of ceramide, a prime sphingolipid signaling molecule, in the pathogenesis of type 1 and type 2 diabetes and its complications. Studies using cultured cells, animal models, and human subjects demonstrate that ceramide is a key player in the induction of β-cell apoptosis, insulin resistance, and reduction of insulin gene expression. Ceramide induces β-cell apoptosis by multiple mechanisms namely; activation of extrinsic apoptotic pathway, increasing cytochrome c release, free radical generation, induction of endoplasmic reticulum stress and inhibition of Akt. Ceramide also modulates many of the insulin signaling intermediates such as insulin receptor substrate, Akt, Glut-4, and it causes insulin resistance. Ceramide reduces the synthesis of insulin hormone by attenuation of insulin gene expression. Better understanding of this area will increase our understanding of the contribution of ceramide to the pathogenesis of diabetes, and further help in identifying potential therapeutic targets for the management of diabetes mellitus and its complications.

## Introduction

Diabetes mellitus is one of the leading causes of death worldwide, ranking amongst cardiovascular diseases and cancer. Diabetes may be classified into two groups based on its pathophysiology. One is caused by autoimmune destruction of β-cells (Type 1 or Insulin dependent diabetes), while the other is due to the progressive decline of pancreatic β-cell function in the context of insulin resistance (Type 2 or Non-insulin dependent diabetes) [1]. The major morbidity threats of diabetes are often microvascular (retinopathy, nephropathy and neuropathy) and macrovascular (cardiovascular diseases) complications [1,2]. According to a recent World Health Organization report, 3.2 million deaths worldwide are attributable to diabetes every year [2]. The number of people with diabetes will be more than double over the next 20 years, reaching a total of 366 million by 2030 [2]. Therefore, a better understanding of the disease and its pathophysiological mechanism is of paramount importance.

Sphingolipids, one of the major classes of lipid within the mammalian lipidome, are characterized by the presence of sphingoid base in their structure [3]. They are ubiquitous constituents of eukaryotic membranes that play a key role in the regulation of signal transduction pathways [4,5]. Deregulation of sphingolipids is implicated in numerous diseases including cancer [6], cardiovascular diseases [7], and neurodenerative disorders [8]. Over the past two decades, accumulating evidence has shown a clear indication that sphingolipids such as ceramide, sphingosine and glycosphingolipids (GSL), have important role in the pathogenesis of both type 1 and 2 diabetes and its associated complications.

The aim of this review is to shed light on the current understanding of the role and mechanism of ceramide in diabetes, and to highlight areas requiring further study. Understanding the complex mechanism of ceramide action in diabetes requires adequate knowledge of insulin signal transduction and sphingolipid biosynthesis. Therefore, the first two sections of this review provide an overview of insulin signaling and sphingolipid metabolic pathways. This review also summarizes the role, and potential cellular signaling mechanisms of ceramide in β-cell apoptosis, insulin resistance, and attenuation of insulin gene expression. This review will also very briefly discuss the role of ceramide in some of the diabetic complications.

### Insulin signaling pathway

Insulin, an endocrine hormone produced from the pancreatic β-cells, plays a key role in glucose homeostasis [9,10]. Insulin action is mediated through insulin receptor (IR) that propagates its activity via three different pathways: phosphatidylinositol-3 kinase (PI3K) pathway, mitogen-activated protein kinase (MAPK) pathway, and Cbl-associated protein (CAP) pathway [11,12]. The PI3K pathway is the major pathway involved in glucose transport, and is significantly distorted by ceramide. The present review specifically focuses on this particular pathway of glucose regulation.

**Figure 1:**
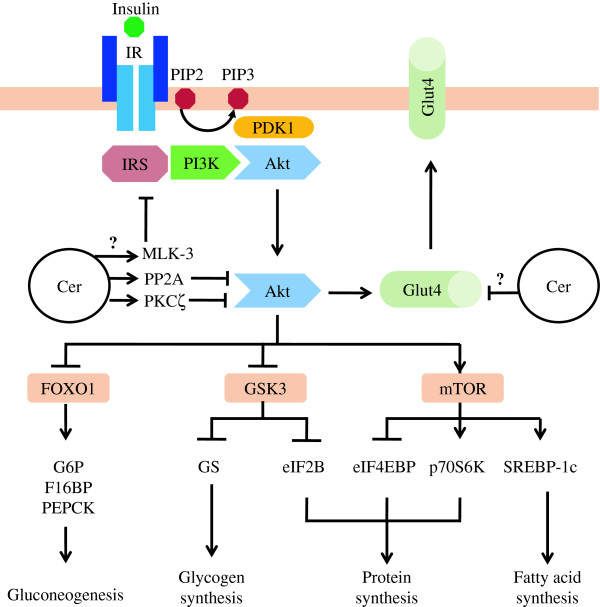
**Major pathways in insulin receptor signaling and mechanism of ceramide in insulin resistance.** Details of these processes and abbreviations are described in the text. Briefly, activation of insulin receptor leads to Akt activation. Once activated, Akt reduce blood glucose level by inducing glucose uptake, glycogen synthesis, protein synthesis and fatty acid synthesis. Akt also act by inhibiting gluconeogenesis. Some of the metabolic effects of insulin may also be mediated via other signaling branches than those depicted. Ceramide causes insulin resistance by PP2A and PKCζ mediated inhibition of Akt and MLK-3 mediated inhibition of IRS. Ceramide may also decrease *Glut-4* gene transcription. Cer, Ceramide.

Insulin receptor is a tyrosine kinase receptor which has two extracellular α-subunits, and two transmembrane β-subunits [11]. Upon insulin binding to the α-subunit, IR undergoes autophosphorylation of tyrosine residues in the intracellular β-domain. Insulin receptor substrate (IRS) has a phosphotyrosine binding domain that recognizes the activated IR, and in turn leads to tyrosine phosphorylation and activation of IRS (Figure 1) [11]. Once activated, IRS allows the binding and activation of PI3K which phosphorylates the membrane lipid phosphatidylinositol (4,5)-bisphosphate (PIP2) to phosphatidylinositol (3,4,5)-triphosphate (PIP3). Protein kinase B (PKB, also known as Akt) is recruited to the plasma membrane and activated at PIP3 site in the presence of phosphoinositide-dependant protein kinase-1 (PDK1) [10]. The activation of Akt allows its relocalization to the cytosol, where it causes the translocation of the glucose transporter-4 (Glut-4) to the plasma membrane, thereby promoting glucose uptake (Figure 1) [13]. Akt also phosphorylates and inactivates glycogen synthase kinase-3 (GSK3), an enzyme involved in glycogen synthase phosphorylation and inactivation. This results in an increase in glucose storage as glycogen [12]. Glycogen synthase kinase-3 is also an endogenous inhibitor of guanine nucleotide exchange factor eIF2B, an essential participant in the initiation of protein translation. Hence, the inactivation of GSK3 by Akt promotes protein synthesis making the amino acids less available for gluconeogenesis [12]. Akt also excludes transcription factor Forkhead box O1 (FOXO1) from the nucleus, abolishing FOXO1 mediated expression of gluconeogenic enzymes such as glucose-6-phosphatase (G6P), fructose-1,6-biphosphatase (F16BP) and phosphoenolpyruvate carboxykinase (PEPCK) (Figure 1) [12,14]. Akt activates mammalian target of rapamycin (mTOR) which promotes protein synthesis through activation of p70 ribosomal S6 kinase (p70S6K), and the inhibition of eIF4E binding protein (eIF4EBP) [15,16]. The activation of mTOR by Akt also promotes fatty acid (FA) uptake and synthesis by activating transcription factor sterol regulatory binding protein 1C (SREBP-1c) which is a protein that enhances the transcription of FA and triglyceride biosynthetic enzymes (Figure 1) [12,14].

### Sphingolipid biosynthesis: regulation and compartmentalization

**Figure 2:**
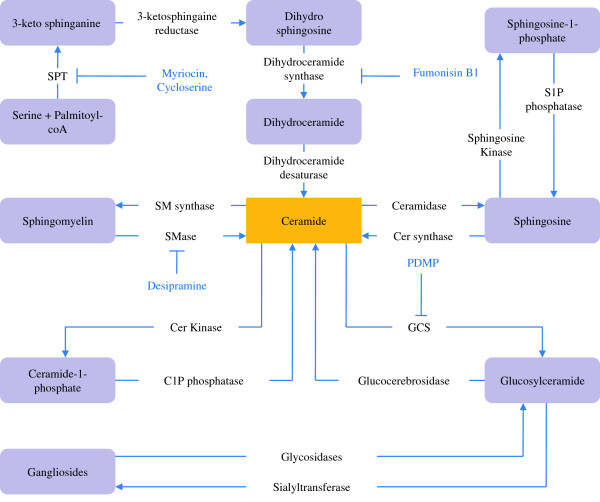
**Regulation of sphingolipid biosynthesis.** Details of these processes and abbreviations are described in the text. Briefly, ceramide is formed from L-serine and palmitoyl CoA via de novo pathway. Ceramide can also be formed from SM hydrolysis by SMase, glucosyl ceramide hydrolysis by Glucocerebrosidase and from sphingosine by ceramide synthase. Sphingolipid metabolites are depicted in box and enzymes are shown outside the box. Some pharmacological inhibitors of sphingolipid biosynthetic enzymes are also shown.

The biosynthetic pathway of sphingolipids consists of a complex network of synthetic and degradative reactions. The *de novo* biosynthesis of sphingolipids occurs at the cytosolic leaflet of the endoplasmic reticulum (ER). During this, 3-ketosphinganine is formed by the condensation of L-serine and palmitoyl CoA by the action of an enzyme serine palmitoyl transferase (SPT). The newly formed 3-ketosphinganine first undergoes rapid reduction to dihydrosphingosine by the action of 3-ketosphinganine reductase, which is then acetylated to form dihydroceramide (dh-Cer) by the action of dh-Cer synthase. The enzyme dh-Cer desaturase reduces dh-Cer to ceramide (Figure 2) [4,5,17]. Ceramide serves as a ‘metabolic hub’ in the sphingolipid metabolic pathway as a substrate for subsequent production of other sphingolipid signaling intermediates [18-20]. Neutral ceramidase in the ER, alkaline ceramidase in the plasma membrane, and acid ceramidase in the lysosome may hydrolyze ceramide to generate sphingosine. Sphingosine may be phosphorylated to sphingosine-1-phosphate (S1P) by sphingosine kinase. The formation of sphingosine from ceramide and S1P from sphingosine can be reversed by enzymes ceramide synthase and S1P phosphatase respectively (Figure 2).

Ceramide once formed in ER, is transported to the Golgi with the help of a transfer protein CERT [20]. The Golgi is the site for the synthesis of sphingomyelin (SM) and glucosylceramide, with the latter serving as the precursor for complex GSLs. An enzyme SM synthase-1 at Golgi, and SM synthase-2 at plasma membrane transfer a phosphorylcholine head group to ceramide and form SM with the release of diacylglycerol (DAG) [4,20]. Alkaline and neutral sphingomyelinase (SMase) at the plasma membrane, and acid SMase at the lysosome can reverse this reaction to generate ceramide back from SM. Glucosylceramide is formed from ceramide by action of membrane bound glucosylceramide synthase (GCS), which can be further converted to complex GSLs by different sialyl transferases. In the lysosome, complex GSLs are degraded back to glucosylceramide, and then to ceramide by the enzymes glycosidases and glucocerebrosidase, respectively [20]. In an alternative pathway, ceramide is phosphorylated at the plasma membrane by ceramide kinase. Its product, ceramide-1-phosphate (C1P), can be hydrolyzed back by C1P phosphatase to generate ceramide (Figure 2) [21].

### Ceramide in pancreatic beta-cell apoptosis

#### Role of ceramide in beta-cell apoptosis

Apoptosis is a programmed death of cells that plays an important role in the maintenance of tissue homeostasis by eliminating harmful or unwanted cells. The mechanism of apoptosis involves complex signaling pathways. The three currently recognized apoptotic signaling pathways are: the extrinsic death receptor, the intrinsic mitochondrial, and the intrinsic ER pathways [22,23]. Literature suggests that the excessive apoptosis of pancreatic β-cell contributes significantly in the pathogenesis of both type 1 [24] and type 2 [25] diabetes. The role of ceramide in β-cell apoptosis in both type 1 and type 2 diabetes is also well established.

Cytokines such as tumor necrosis factor-alpha (TNF-α), Interleukin*-*1 beta (IL-1β) and Interferon*-*gamma (IFN-γ) are known to exert cytotoxic effects on pancreatic β-cells [26]. Emerging evidence suggest that ceramide has a role in β-cell apoptosis induced by these cytokines [27]. Exposure of insulin-producing MIN6 cells and RINm5F cells to TNF-α and IL-1β, respectively, results in an increase in ceramide production. In addition, ceramide, either delivered exogenously or generated endogenously, mimicked the cytotoxic effect of TNF-α and IL-1β in these cell lines [28-30]. On the other hand, another β-cell model, β-TC3 cells did not show an increase in ceramide when treated with cytokines [31]. This disparity may be due to an immediate activation of ceramidase in β-TC3 cells as reported with INS-1 β-cells [32]. Ceramidase is capable of converting the ceramide to sphingosine which can be further converted to S1P by sphingosine kinase. This is further evidenced with the presence of enhanced S1P level in INS-1 cells following treatment with IL-1β and TNF-α [33]. Such a possibility has to be tested in β-TC3 cells employing sensitive assay methods.

Long-standing evidence implicates that prolonged exposure to free fatty acid (FFA) has detrimental effects on pancreatic β-cells. Ceramide has been proposed to be a mediator of FFA-induced β-cell toxicity. Palmitate, a precursor of ceramide has been found to induce islet cell apoptosis in diabetic rat models, healthy rats, and human β-cells [34-36]. However, unsaturated FA failed to induce this effect [35,37,38]. Similarly, studies conducted in Zucker diabetic fatty (ZDF) rats demonstrated that ceramide formed via the *de novo* pathway is responsible for the FFA-induced β-cell apoptosis. This is evidenced with the appearance of marked increase in [^3^H]-ceramide up on culturing islet cells in the presence of either [^3^H]-serine or [^3^H]-palmitate [34,39]. In addition, inhibition of ceramide synthesis using SPT inhibitor (L-cycloserine) or ceramide synthase inhibitor (fumonisin-B1), has been reported to attenuate FFA-induced cytotoxicity in both rodent [34,35,39] and human [37,40] β-cells. In contrast, some studies with β-cell apoptosis have reported only modest increase in ceramide upon treatment with FFA. However, inhibition of *de novo* ceramide synthesis reduced the apoptosis significantly [41]. There could be three possible explanations for this contradictory result. First, an increase in ceramide at particular cellular location, without altering the total ceramide mass may be sufficient to induce apoptosis. This is supported by the fact that ceramide synthesized in the ER can be transported to other organelles including mitochondria, and moreover, mitochondria itself contains the enzymatic machinery necessary for ceramide synthesis [42]. A second possible explanation is conversion of ceramide to another sphingolipid metabolite after apoptotic induction. To support this, Boslem et al. identified an increase in glucosyl ceramide, a metabolite of ceramide up on treatment of MIN β-cells with FFA. They also found that the overexpression of glucosyl ceramide synthase, an enzyme that converts ceramide to glucosyl ceramide, did not exacerbate, but partially protected from apoptosis [43]. Finally, apoptotic potential of ceramide may vary with different isoforms. Veret et al. demonstrated that FFA, at low glucose concentration, increased all ceramide species, but at elevated glucose concentration increased the more toxic C18:0, C22:0 and C24:1 isoforms of ceramide specifically to enhance apoptosis [44]. Conclusive data from these studies demonstrate that ceramide generated via the *de novo* pathway is the key player in the execution of FFA-induced β-cell apoptosis.

#### Mechanism of ceramide induced beta-cell apoptosis

##### Activation of extrinsic apoptotic pathway

**Figure 3:**
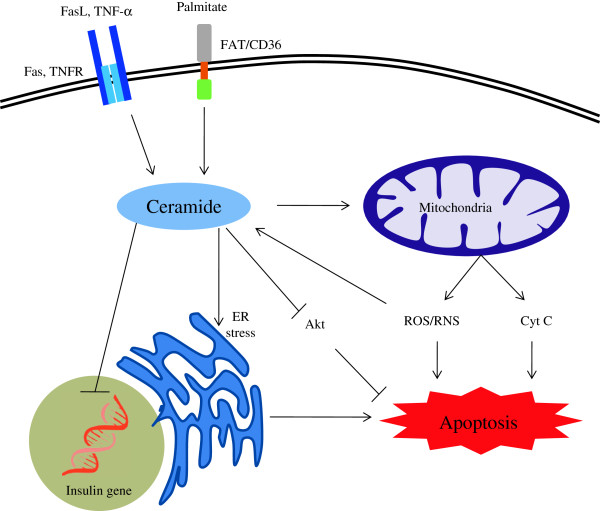
**Schematic representation of mechanism of beta-cell apoptosis caused by ceramide.** Details of the processes and abbreviations are described in the text. Briefly, cellular ceramide can be formed by de novo biosynthesis from precursor palmitate and by the activation of extrinsic pathway of apoptosis by FasL or TNF-α binding to the Fas and TNF receptor (TNFR) respectively. Ceramide acts on the mitochondria and causes the release of ROS/RNS and cytochrome c and activates intrinsic apoptotic pathway. Increased ROS can further increase ceramide generation. Ceramide can act on the ER and cause ER stress mediated apoptosis. Ceramide also inhibits insulin gene expression.

It has been generally accepted over the past few decades that activation of the extrinsic pathway of apoptosis plays a significant role in β-cell loss. The extrinsic pathway begins outside the cell with the binding of death ligands, such as TNF-α and Fas ligand, to their respective cell-surface death receptors such as TNF receptor and Fas (CD95). This binding of the receptor by its cognate ligand results in the recruitment and activation of initiator caspase (mostly caspase-8), which then propagates apoptosis by cleaving and activating downstream effector caspases such as caspase-3 [45,46]. Agent that induces ceramide accumulation en route to β-cell apoptosis, such as cytokines and saturated FAs, were also reported to activate the extrinsic pathway of apoptosis [27,35] (Figure 3). Normal pancreatic β-cells exhibit only minimal expression of Fas, but when exposed to cytokines, such as IL-1, an increase in Fas expression was observed [47]. Pancreatic β-cell that lacks caspase-8 has shown protection against both Fas and ceramide-induced cell death, suggesting the role of ceramide in it [48]. Moreover, Liadis et al. demonstrated that caspase-3 knockout mice protected from developing diabetes [49], signifying the role of caspase cascade in β-cell apoptosis.

##### Alteration of mitochondrial membrane permeation

Ceramide induces β-cell apoptosis by increasing the mitochondrial membrane permeability, leading to the activation of the intrinsic pathway of apoptosis. The intrinsic mitochondrial pathway is initiated within the cell in response to a wide range of death stimuli. Regardless of the stimuli, pro-apoptotic molecule, such as cytochrome c, is released into the cytosol which together with another pro-apoptotic protein, Apoptotic protease activating factor 1 (Apaf-1) activates the initiator caspase (mostly caspase-9). The initiator caspase then activates an effector caspase such as caspase-3 that propagates the apoptotic signal [50]. The intrinsic pathway is regulated by a group of proteins belonging to the B-cell lymphoma-2 (Bcl-2) family. There are two main groups of the Bcl-2 proteins, namely the pro-apoptotic proteins (e.g. Bad, Bid, Bax, Bak, Bcl-Xs, Bim, etc) and the anti-apoptotic proteins (e.g. Bcl-2, Bcl-xL, Mcl-1, etc). The anti-apoptotic proteins reduce apoptosis by blocking the mitochondrial release of cytochrome-c, while the pro-apoptotic proteins act by promoting it. The fate of the cell is determined by the tilt in their ratio towards one or the other [46,50].

Ceramide either delivered exogenously or generated endogenously when targeted to the mitochondria, an increase in its permeability to cytochrome c and apoptosis is observed [51,52] (Figure 3). The apoptotic action of ceramide is mediated by the recruitment and activation of pro-apoptotic Bax at the mitochondria. Birbes et al. [53] and Kashkar et al. [54], demonstrated that induction of SMase, an enzyme that converts SM to ceramide, promotes the translocation and activation of Bax at the mitochondria which in turn leads to the release of cytochrome c [55]. In agreement with this, depleting the Bax or antagonizing its activity by overexpressing Bcl-2, prevented ceramide-induced apoptosis [56,57]. The intrinsic mitochondrial pathway is also involved in the FFA-induced β-cell apoptosis. It has been reported that exposure of β-cells to saturated FAs results in enhanced mitochondrial membrane permeability and cytochrome c release [58]. Furthermore, reduction in Bcl-2 expression [36] and Bax up-regulation [58] was also observed when β-cells were exposed to FFAs. Jointly, these studies suggest that ceramide generated from FFA may very well be involved in the activation of the intrinsic apoptosis pathway. Yet, another mechanism of ceramide in mitochondrial membrane alteration has also been proposed. Siskind et al. proposed that ceramide is directly capable of forming channels in the mitochondrial membrane, thus, increasing its permeability to cytochrome c [59]. This action of ceramide has not been extensively studied yet. Ceramide also interferes with Akt activation, and thereby, promotes the mitochondrial membrane permeability, and cytochrome c release by preventing the Akt-induced inactivation of pro-apoptotic Bcl-2 members [60].

##### Free radical generation

Reactive oxygen species (ROS) and reactive nitrogen species (RNS) are some of the key integrating mediators in the development of β-cell apoptosis. Although, relevant amount of ROS and RNS are essential for maintaining normal physiological signaling and metabolic functions, excessive amount of these radicals can lead to cell death and tissue damage [61]. Ceramide plays an important role in ROS and RNS generation through activation of nicotinamide adenine dinucleotide phosphate (NADPH) oxidase [62], initiation of mitochondrial dysfunction [63], induction of inducible nitric oxide synthase (*iNOS*) gene expression [64], and down-regulation of anti-apoptotic Bcl-2 proteins [65]. Conversely, a growing body of evidence also suggests that ROS and RNS enhance ceramide generation by inducing SMase [66], or inhibiting ceramidase [67] enzymes (Figure 3). Although, these effects are not extensively studied in the pancreatic β-cells, the bidirectional interaction of ceramide and ROS/RNS may also be involved in the apoptotic cascades in β-cells.

The major source of ROS in most cells is the leakage of electrons from complex I and complex III of the mitochondrial respiratory chain [14]. Inhibition or reduction of activities of these complexes results in electron leakage, and elevated ROS level which in turn can lead to β-cell apoptosis. Ceramide has been shown to disrupt electron transport at complex I [68] and complex III [69], resulting in an enhanced ROS generation which facilitates cytochrome c release and caspase activation [63]. Similarly, TNF-α, a known inducer of ceramide is also found to inhibit complex III activity [14]. As described earlier, activation of NADPH oxidase by ceramide also leads to ROS generation and apoptosis if it is not rapidly removed by the antioxidant defense [62]. The exact mechanism of ceramide-mediated NADPH oxidase activation is not known, but it is believed that ceramide activates protein kinase C zeta (PKCζ), which facilitates binding of p67^phox^, a catalytic subunit of NADPH oxidase, to its holo enzyme complex [70,71]. Another mechanism of ceramide-induced free radical generation is via regulation of anti-apoptotic Bcl-2 protein. Bcl-2 prevents ROS production, increases antioxidant GSH pool and redistributes it [65]. In islet cells, ceramide is reported to decrease Bcl-2 mRNA expression [72]. Yet, another mechanism of ceramide-induced free radical generation is via increased expression of *iNOS*, which codes for iNOS enzyme involved in the synthesis of nitric oxide (NO) in response to inflammatory stimuli such as cytokines and lipotoxicity. Ceramide, either generated via the *de novo* pathway [73] or SM hydrolysis [74], is reported to induce nuclear factor kappa B (NFκB), a transcription factor involved in the expression of *iNOS* gene activation. When islets of ZDF rats were supplied with FAs, an increase in apoptosis along with increased *iNOS* expression and nitric oxide formation was observed [34]. These effects were blocked by fumonisin B1 and aminoguanidine, a NOS inhibitor [34], suggesting the role of ceramide in it. Finally, ROS also acts by inducing ER stress that further induces β-cell apoptosis [75]. In conclusion, all these studies suggest that ROS/RNS generation triggered by ceramide may be one of the crucial factors in promoting apoptosis in many mammalian cells and animal models, but further studies are required to understand the complete involvement of these free radicals in β-cell apoptosis.

##### Induction of ER stress

Accumulating evidence suggests that ER stress plays an important role in β-cell apoptosis [76,77]. During diabetes there will be high secretory demand for insulin placed on the pancreatic β-cells to counteract hyperglycemia. The ER, being the site of synthesis and folding of secreted proteins, is highly susceptible to stress when the demand for insulin folding and secretion exceeds its capacity [78,79]. This increased ER stress leads to accumulation of misfolded and unfolded insulin in the ER, which may activate Unfolded Protein Response (UPR) to restore the normal ER function. When restoration fails, UPR switches in to an alternative mode and turns on the apoptotic signaling pathway in the β-cells. Oyadomari et al. and Socha et al. demonstrated ER stress-induced β-cell apoptosis in diabetic animal models [80,81]. Similarly, elevated levels of ER stress markers were observed in islets of diabetic human subjects [82]. Fatty acids, particularly saturated FAs have been shown to promote ER stress to induce β-cell apoptosis [82,83]. This specific toxic effect of saturated FAs may be related to the formation of ceramide from it [41]. Collectively, observations from several studies demonstrate that ceramide synthesis via the *de novo* pathway is involved in ER stress-induced β cell apoptosis [43,44]. In line with this, ceramide generated via SM hydrolysis has also been reported to cause ER stress-induced β-cell apoptosis. Lei et al demonstrated that activation of Ca2 + -independent phospholipase A2 (iPLA2β), an inducer of β-cell apoptosis in response to strong ER stress, promotes ceramide accumulation secondary to the activation of neutral SMase, an enzyme that hydrolyzes SM to generate ceramide [84]. Inhibition of neutral SMase protected β-cell from ER stress induced apoptosis, demonstrating the importance of ceramide in ER stress-induced cell death. Ceramide generated by ER stress also activates intrinsic mitochondrial pathway of apoptosis in β-cells by altering the mitochondrial membrane permeability and release of cytochrome c [84]. ER stress and induction of UPR can also result in ROS generation, and the role of ROS, both as upstream and downstream, to ceramide activation is well known [85].

##### Inhibition of Akt

A major mechanism through which ceramide induces β-cell apoptosis is by its inhibitory action on Akt, a serine/threonine kinase which regulates several biological processes including cellular growth, proliferation and survival in multiple organs (Figure 3). Akt mediates its proliferative and anti-apoptotic action on the pancreatic β-cell through several mechanisms. First, Akt phosphorylates and induces cytosolic retention of cyclin-dependent kinase inhibitors (CKI) such as p21Cip1 and p27Kip1. This enhances the proteosomal degradation of these CKIs [86]. Second, Akt negatively regulates the transcriptional activity of FOXO1, which is known to upregulate p27kip1 [86]. Third, Akt directly phosphorylates and inactivates pro-apoptotic Bcl-2 members such as Bad, Bax and Bid [87]. Finally, Akt activates mTOR/p70S6K mediated cell growth and proliferation [15,16]. Through all these mechanisms, Akt promotes the cell cycle, proliferation and inhibition of apoptosis.

An inverse correlation between ceramide and Akt activation has been reported in cultured cells when exposed to ceramide [12,15,16,88-90]. Akt inactivation along with ceramide accumulation is also observed in rats treated with glucocorticoids and saturated fat [91]. Inhibition of ceramide biosynthesis using myriocin, cycloserine, or fumonisin B1 restored the Akt activity [15,91,92]. As an alternative strategy to manipulate endogenous ceramide, cultured cells when treated with PDMP, an inhibitor of ceramide glucosylation, exacerbated palmitate-induced Akt inactivation [93]. This palmitate effect was reversed by over expressing acid ceramidase [92,94]. These studies support the hypothesis that Akt inactivation by ceramide is one of the contributing mechanisms by which ceramide causes β-cell apoptosis. The mechanism through which ceramide inhibits Akt activity will be discussed later in this review.

### Ceramide in insulin resistance

#### Role of ceramide in insulin signaling and action

It is becoming increasing apparent that ceramide plays significant role in insulin resistance, a metabolic state in which cells fail to respond to the normal hormonal actions of insulin. Evidence from various studies revealed that excess FFA intake, glucocorticoid administration, obesity, and lack of physical exercise are some of the important causations leading to insulin resistance. Plethora of studies using wide variety of cultured cells, animal models and human subjects demonstrated ceramide as the key intermediate linking all these conditions to insulin resistance [95].

Preliminary evidence underpinning the role of ceramide in insulin resistance came from the direct application of ceramide to isolated skeletal muscles and cultured adipocytes. These studies indicate that ceramide inhibits insulin-stimulated glucose uptake and glycogen synthesis [96,97]. Increased delivery of saturated FA in excess of a tissue’s oxidative or storage capacity is one of the main reasons for insulin resistance. Prolonged exposure of palmitate to cultured myotubes [15], L6 skeletal muscle cells [16], 3 T3-L1 adipocytes [98] and cardiac myocytes [99] increases ceramide accumulation with simultaneous inhibition of Akt. Consistent with this, palmitate exposure also caused a reduction in glucose uptake and glycogen synthesis [92]. Subsequent studies using pharmacological inhibitors or small interfering RNA (siRNA) to block the enzymes involved in ceramide biosynthesis, have demonstrated that ceramide is an obligate intermediate in saturated FA-induced insulin resistance [94,100,101]. In agreement with this, overexpression of acid ceramidase as an alternative approach to reduce the ceramide level, negated the palmitate-induced ceramide accumulation and improved insulin signaling [93]. Similarly, acute application of ceramide analogue to 3 T3-adipocytes mimicked the palmitate induced insulin desensitizing effect [102].

As mentioned earlier, some groups also studied the role of saturated FA in insulin resistance using animal models, including high fat-fed mice, ob/ob mice, lipid-infused rats, dexamethasone-treated rats and ZDF rats [103-105]. Recently, Frangioudakis et al. demonstrated that mice fed with high-fat diet shows increased expression of ceramide synthase [103]. Infusion of lipid emulsion in animal model has shown to increase muscle ceramide content and decrease peripheral insulin sensitivity [105]. These effects were blocked by using SPT inhibitors, indicating the role of ceramide [105]. Recently, Holland et al. compared the effect of lard oil (high in saturated FAs) and soy oil (high in unsaturated FAs) on insulin sensitivity in rats. They found that both of these treatments decreased glucose uptake and Akt activation, but ceramide increase was observed only with lard-oil infusion [105]. Similarly, several other studies also could not find considerable increase in ceramide in response to lipid supplementation, but an elevated DAG level was observed [106,107]. When carefully examined, some of these studies were found to use lipid rich in unsaturated FA, indicating that saturated fats and unsaturated fats have different mechanisms of promoting insulin resistance, and ceramide plays a role only in insulin resistance induced by saturated fats [105]. As mentioned earlier, the experiments conducted to study the effect of FA on ceramide generation and insulin resistance, were not only restricted to cell lines and animal models. Studies conducted in human subjects also provided precious information. Studies conducted using insulin-resistant human subjects demonstrated almost two fold increase in ceramide accumulation compared to normal subjects [108]. In another study, lipid infusion in humans is found to increase skeletal muscle ceramide and decrease insulin sensitivity [109].

Apart from inducing ceramide generation, chronic exposure to elevated concentrations of FAs have been reported to modulate several other effectors and signaling pathways, to induce beta-cell dysfunction and apoptosis. Some of those effectors include endocannabinoids, eicosanoids, cytokines, and transcription factors. Endocannabinoids are FA derivatives implicated in the regulation of energy balance, hepatic lipogenesis, and glucose homeostasis [110,111]. Elevated levels of endocannabinoids, such as 2-arachidonoylglycerol or anandamide, have been implicated in hyperglycemia and decreased insulin sensitivity in high fat fed mice and obese human subjects [111]. Inhibition of endocannabinoid activity, using endocannabinoid receptor antagonist, resulted in increased glucose uptake in diabetic mice models [111]. Eicosanoids are another class of FA derivatives that play vital role in the control of pancreatic β-cell function and survival [112,113]. Cyclooxygenase derived eicosanoids, such as prostaglandin E2 and eicosanoids of 12-Lipoxygenases, have been found to attenuate glucose-stimulated insulin secretion and increase beta-cell destruction in the pancreas [113]. NF-κB is a transcription factor which is best known for its immune and inflammatory responses. Key role of the NF-kB pathway in the induction of inflammatory responses, that underlie type 2 diabetes, has been highlighted in several studies [114,115]. Fatty acids, in particular saturated FAs induce the expression of NF-κB through Toll-like receptor 4 (TLR4) signaling [115]. An improvement in insulin sensitivity was observed in animal model of FFA-induced insulin resistance when the gene encoding for TLR4 was mutated [115].

Glucocorticoids are most commonly used therapeutic agents, although contraindicated in diabetes as they can cause insulin resistance [116]. Previously, it has been demonstrated that glucocorticoids induce ceramide generation which may be responsible for its induction of insulin resistance [105]. Dexamethasone, a widely used glucocorticoid was shown to increase ceramide level in broad range of cell types, and animal subjects through stimulation of enzymes such as SPT, SMase and ceramide synthase [105,117,118]. Some of this effect was completely prevented by pre-treating the subjects with myriocin [105]. It has been reported that glucocorticoid-induced ceramide accumulation and associated insulin resistance requires activation of peroxisome proliferator activated receptor (PPAR) alpha. As an evidence to this, genetic ablation of PPAR alpha, or disruption of hepatic vagal nerves (which decreases hepatic PPAR alpha expression) prevented dexamethasone-induced insulin resistance [119,120]. Thiazolidinediones (TZDs) are most commonly used anti-diabetic agent which functions as insulin sensitizers [121]. It is found that TZDs like pioglitazone [122], troglitazone [123], and rosiglitazone [124] decrease ceramide accumulation in muscles of rat or mice. Fenretinide, a chemotherapeutic agent is found to improve insulin sensitivity in high fat-fed mice, was recently identified as an inhibitor of dihydroceramide desaturase. Thus, insulin-sensitizing actions of some of these drugs may result from its inhibitory effects on ceramide synthesis [125].

Physical exercise is widely perceived to be beneficial for glycemic control in type 2 diabetes, as it improves insulin sensitivity and glucose homeostasis [126]. Routine exercise training is found to decrease ceramide content in skeletal muscles of rat, mice [127,128] and human subjects [129,130]. In contrast, some studies could not report significant decrease in muscle ceramide level even after exercise training in rats and humans [131,132]. The reason for this discrepancy remains unclear. Obesity and associated increase in pro-inflammatory cytokines is another key player of insulin resistance [133]. Patients with obesity have elevated level of the inflammatory cytokine such as TNF-α [134]. The role of TNF-α in ceramide generation has already been discussed in this review. Finally, insulin resistance increases with age. Wu et al. demonstrated that adipocytes from older mice contained higher ceramide compared to younger one [135]. Conclusive evidence from all these studies suggests that inhibitors of ceramide synthesis or activators of ceramide degradation may prove efficacious as therapeutics to combat insulin resistance.

#### Mechanism of ceramide-induced insulin resistance

The precise molecular mechanisms by which changes in ceramide might cause insulin resistance are not entirely clear. Although, it seems likely that ceramide influences several distinct intermediates in the insulin signaling pathway. Some of these potential mechanisms by which ceramide may impair insulin signaling and action are discussed below.

##### Ceramide on IRS

Kanety et al. reported that ceramide inhibits insulin stimulated tyrosine phosphorylation of IRS-1 [136]. This is confirmed by some other studies which demonstrated that ceramide phosphorylates inhibitory serine residues on IRS-1 [136,137]. The probable mechanism is that ceramide activates mixed lineage kinase-3 [138] (Figure 1), which in turn activates stress-activated protein kinases such as p38 and c-Jun N-terminal kinase (JNK) [139,140]. These enzymes have been implicated in phosphorylation of serine-307 on IRS-1 [141,142]. This in turn inhibits the necessary tyrosine phosphorylation needed for insulin signal transduction [143]. In agreement to this, Hirosumi et al. demonstrated an increase in JNK activity and serine-307 phosphorylation, and a decrease in tyrosine phosphorylation of IRS-1 in tissues of obese mice [142]. In another study, mutations in the gene coding for JNK-binding protein (an inhibitor of JNK activity) in humans caused type 2 diabetes [10,144]. Even though these studies demonstrated ceramide-induced inhibition of IRS-1, other studies did not find any correlation [16,92].

##### Ceramide on PI3K, PDK1, Phosphoinositides (PIP2 and PIP3) and Glut-4

Several studies evaluated the role of sphingolipids on PI3K [16,91,92], PDK1 [145], phosphoinositide (PIP2 and PIP3) [146,147] and Glut-4 [98]. However, majority of these studies failed to see any direct effect. Although Zundel et al. reported that ceramide inhibits PI3K activity [148], yet, its relevance in the regulation of glucose homeostasis remains unclear.

##### Ceramide on Akt/PKB

The role of ceramide in regulating the Akt to induce β-cell apoptosis has been discussed in the preceding section of this review. Indeed, the involvement of Akt in β-cell physiology might go beyond the induction of apoptosis and include the regulation of insulin secretion. Therefore, the inhibition of Akt by ceramide might have a negative impact on insulin sensitivity as well by abrogating all Akt mediated insulin activities (Figure 1).

Inhibition of Akt activation by ceramide is thought to be accomplished by at least two mechanisms. First, ceramide activates protein phosphatase 2A [92,149] (Figure 3) which catalyses the dephosphorylation of Akt by removing activating phosphates [92,145,146,150]. The Akt inhibitory effect of ceramide in cell lines like PC12 cells [145], C2C12 myotubes [92], human glioblastoma cell [150], and brown adipocytes [149], was negated by the PP2A inhibitor, Okadaic acid. When PP2A activity was impaired by over expressing SV40 small T antigen (which displaces the regulatory subunits that target PP2A to its substrates), the effect of ceramide on Akt was blocked [99]. Second, ceramide blocks insulin-stimulated Akt translocation to the PIP3-PDK1 complex at the plasma membrane [16,147]. Powell et al. and Bourbon et al demonstrated that ceramide binds to cysteine rich ceramide binding domain on PKCζ and activates it [94,151,152] (Figure 3). The activated PKCζ in turn phosphorylates inhibitory the serine or threonine residue (depending on the Akt isoforms) at the site 34 in the pleckstrin homology (PH) domain of Akt [152]. This prevents its interactions with PIP3, may be by forming more stable Akt-PKCζ complex [94,152]. In agreement with this mechanism, PKCζ inhibitors were found to increase insulin sensitivity and prevent ceramide-induced loss of Akt activation [152,153].

#### Ceramide in lipid raft and diabetes

**Figure 4:**
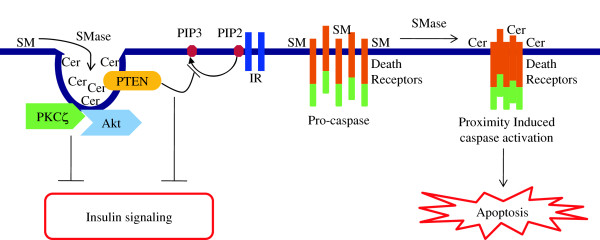
**Schematic representation of role of ceramide in lipid raft and diabetes.** Details of the processes and abbreviations are described in the text. Briefly, the SM within the raft can be hydrolyzed by SMase to form ceramide which immediately self-associate and form ceramide enriched membrane domains. This raft coalescence facilitates apoptosis by initiating proximity induced caspase activation. Ceramide recruits both PKCζ and Akt to the caveolae. By doing so, ceramide can not only activate PKCζ, but also causes co-localization of activated PKCζ with Akt, there by suppressing Akt activity. Ceramide also recruits PTEN in the caveolae which dephosphorylate PIP3 and impair insulin signaling. Cer, Ceramide.

Lipid rafts are specialized micro-domains of plasma membrane that contain high concentrations of lipid derived molecules such as cholesterol, sphingolipids and a subset of phospholipids [154]. Sphingomyelin is the most prevalent sphingolipid present in the outer leaflet of the plasma membrane and hence in the lipid raft. The SM within the raft can be hydrolyzed by SMase and lead to the generation of ceramide [155] (Figure 4). Previously discussed key players of diabetes induction such as TNF receptor activation, Fas activation and ROS generation were reported to activate SMase and hence generate ceramide in the rafts [156]. Ceramide molecules associate with each other and immediately form small ceramide-enriched membrane microdomains. The generation of ceramide and its tight packing within rafts dramatically alters the structure and composition of these domains. This results in spatial re-organization and clustering of several cytokine and death receptors, including Fas and TNF receptors [155,156]. This receptor clustering perhaps could promote β-cell apoptosis through proximity induced caspase activation (Figure 4). In line with this, cells that lack SMase activity failed to release ceramide and induce apoptosis upon Fas and TNF receptor stimulation [157]. Consistent with this, neutralization of ceramide in ceramide-enriched membrane domains using anti-ceramide antibodies inhibited Fas-induced apoptosis [158]. However, additional experimental evidence is required to elucidate the entire role of receptor clustering in diabetes.

Caveolae are a subset of lipid rafts seen as flask like invaginations on the plasma membrane [159]. Apart from ceramide, these structures are characterized by the presence of proteins termed caveolin, a family of scaffolding proteins which play significant role in numerous signaling pathways by compartmentalizing and concentrating signaling molecules [159]. Generally, optimal level of caveolae is important for the proper insulin signaling. However, excessive caveolae is reported to impair insulin signaling and action, basically via two mechanisms. First, ceramide extensively recruits and elevates phosphatase and tensin homolog deleted on chromosome 10 (PTEN) in caveolae. The anti-proliferative PTEN specifically catalyses the dephosporylation of the 3′ phosphate in PIP3 and convert it to PIP2 (Figure 4). This decrease in PIP3 in turn, impairs insulin signaling because it is essential for the activation of Akt in response to the IR activation by insulin [160,161]. Second, ceramide recruits both PKCζ and Akt to the caveolae which will have synergetic repressive effect on insulin signaling. Briefly, recruitment of both PKCζ and Akt by ceramide leads to the collective presence of ceramide, PKCζ and Akt in the caveolae [153,161] (Figure 4). By doing so, PKCζ would not only be exposed to ceramide rendering it active, but at the same time would be co-localized with Akt, there by suppressing its activity. Both of these mechanisms together create a highly repressive environment for Akt, and hence, diminish insulin mediated signaling response.

#### Ceramide and insulin synthesis

Thus far, we have mainly considered the role for ceramide in inducing β-cell apoptosis and insulin resistance. However, several studies reveal that ceramide also diminishes insulin synthesis by decreasing the insulin mRNA levels in islets. In pancreatic β cells, saturated FA has been shown to impair insulin gene expression with associated increase in ceramide [162,163]. This effect was largely prevented by inhibitors of *de novo* ceramide synthesis suggesting the role for ceramide [163]. Furthermore, addition of exogenous ceramide or elevation of endogenous ceramide, using ceramidase inhibitor, has been demonstrated to reduce insulin mRNA levels [163,164]. Two postulations have been made to explain the mechanisms of inhibition of insulin gene transcription by ceramide. First, ceramide activates JNK which inhibits insulin gene transcription both via c-jun-dependent [165], and oxidative stress-dependent [166] pathways. Second, ceramide directly activates PKCζ [167] which phosphorylates and inactivates Pancreatic and duodenal homeobox gene-1 (*PDX-1*), a transcription factor which regulates insulin gene expression [168]. Apart from down-regulating insulin gene transcription, ceramide has also been shown to decrease transcription of the *Glut-4* gene [169] (Figure 1). In conclusion, these results suggest that ceramide modulates signaling pathways implicated in the transcriptional regulation of the insulin gene. However, further investigation is required to understand the exact mechanism of inhibition of insulin gene expression by ceramide.

#### Ceramide in diabetic complications

The major threats of diabetes are its serious, sometimes life-threatening complications. A growing body of evidence has identified the role of sphingolipids, particularly ceramide, in the pathogenesis of both microvascular and macrovascular diabetic complications. Cardiovascular diseases such as atherosclerosis, myocardial infarction and stroke, are major cause of mortality in diabetic patients. Diabetic cardiomyopathy is characterized by the apoptosis of cardiomyocytes [170]. Increased myocardial ceramide content was observed in various rodent models of lipotoxic cardiomyopathy [171,172]. *De novo* ceramide biosynthesis, when inhibited either pharmacologically by myriocin, or genetically by heterozygous deletion of SPT subunit, an improvement in the cardiac function was observed [171,172]. Apart from this, Gorska et al. demonstrated an elevated acid SMase level in plasma of type 2 diabetic patients [173]. Ceramide has also been implicated in atherosclerosis in both human subjects and in animal models [174,175], and regression of atherosclerotic plaques was observed when treated with myriocin [176]. Conclusively, these studies suggest that ceramide generated via *de novo* pathway and SM hydrolysis may be involved in the development of diabetic cardiovascular complications.

Nephropathy represents another major threat in diabetic patients which is characterized by the apoptosis of renal mesangial cells. Several studies reported the role of ceramide in mesangial cell apoptosis. Increased expression of SPT was seen in renal tubular epithelial and microvascular endothelial cells, which are the main sites of apoptosis observed in diabetic patients [177]. When ceramide generation was inhibited using SPT inhibitors and ceramide synthase inhibitors, a reduction in tubular epithelial cell death was observed [178,179]. Another important complication associated with diabetes is development of neuropathy. Except for the gangliosides, role of sphingolipids in diabetic neuropathy is not extensively studied. When mouse Schwann cells were cultured with palmitate, an enhancement in apoptosis was observed. This effect was significantly suppressed by myriocin and fumonisin B1, suggesting a role for ceramide [180]. Another important complication of diabetes is retinopathy, the second leading cause of blindness in the developed countries [181]. When cultured retinal pericytes were incubated with palmitate, an increase in cellular ceramide content with subsequent increase in apoptosis was observed [182]. This effect was reversed by overexpression of ceramidase [182]. In another study where ceramide accumulation was induced by using Advanced Glycation End products, Fumonisin B1 did not reverse the retinal pericyte apoptosis, suggesting that *de novo* biosynthesis is not involved in the ceramide generation [183]. Here, desipramine, an acid SMase inhibitor, almost completely abolished ceramide generation and apoptosis, demonstrating that ceramide is formed from SM hydrolysis [183].

These observations underline the potential involvement of ceramide in the pathogenesis of diabetic complications. Moreover, some of the studies also demonstrated the role of other sphingolipids such as GSL in diabetic nephropathy [184] and retinopathy [185]. However, whether it is ceramide or GSL that play a more important role in these diabetic complications is not known. Further research employing more advanced lipidomic screening will definitely bring the answer to this question.

## Conclusion

There has been considerable progress over the last few years in unraveling the role and mechanism of ceramide action in diabetes and its complications. Taken together, evidence from a plethora of studies indicates that ceramide plays a significant role in diabetes by at least three different mechanisms: inducing pancreatic β-cell apoptosis, increasing insulin resistance, and reducing insulin gene expression. Translated to the clinical level, all of these ceramide mediated pathways remain candidates for their putative contributions to the pathogenesis of diabetes. However, there are still several unanswered questions. First, it is not known what enables ceramide to mediate so many diverse signaling events in the cell. Though different species of ceramide exist, the role and critical importance of each species in the pathophysiology of diabetes is not as yet completely known. May be this species diversity is responsible for the multiple cellular events mediated by ceramide. Secondly, Ceramide generated in the ER can be transported to different organelles, and moreover, many organelles contain the machinery that enables them to synthesize ceramide. Hence, the quantitative and qualitative aspects of ceramide in different sub-cellular compartments must be extensively studied. Third, sphingolipids have highly interconnected metabolic networks, so alteration of ceramide might have deleterious impact on other sphingolipid metabolites. The impact of such modifications on the normal functioning of cells must be studied. Finally, the factors that regulate sphingolipid biosynthetic enzymes to generate ceramide during diabetes induction have to be investigated extensively. Further elucidation of these molecular details will be essential to develop better understanding of the validity of ceramide modulation as a strategy for treating diabetes. Advances in analytical lipidomics such as tandem mass spectrometry and lipid imaging could provide more crucial information regarding the role of ceramide in the etiology and pathogenesis of diabetes in the coming years. Such an understanding undoubtedly will have a direct impact on future therapies for diabetes.

## Abbreviations

Apaf-1: Apoptotic protease activating factor 1; Bcl-2: B-cell lymphoma-2; C1P: Ceramide-1-phosphate; CAP: Cbl-associated protein; CKI: Cyclin-dependent kinase inhibitors; DAG: Diacylglycerol; dh-Cer: Dihydroceramide; eIF4EBP: elF4E binding protein; ER: Endoplasmic reticulum; F16BP: Fructose-1,6-biphosphatase; FA: Fatty acid; FFA: Free fatty acid; FOXO1: Forkhead box O1; G6P: Glucose-6-phosphatase; GCS: Glucosylceramide synthase; Glut4: Glucose transporter-4; GSK3: Glycogen synthase kinase-3; GSL: Glycosphingolipid; IFN-γ: Interferon*-*gamma; IL-1β: Interleukin*-*1 beta; iNOS: inducible nitric oxide synthase; iPLA2β: Ca2 + -independent phospholipase A2 beta; IR: Insulin receptor; IRS: Insulin receptor substrate; JNK: c-Jun N-terminal kinase; MAPK: Mitogen-activated protein kinase; mTOR: mammalian target of rapamycin; NADPH: Nicotinamide adenine dinucleotide phosphate; NFκB: Nuclear factor kappa B; NO: Nitric oxide; p70S6K: p70 ribosomal S6 kinase; PDK1: Phosphoinositide dependant protein kinase-1; PDX-1: Pancreatic and duodenal homeobox gene-1; PEPCK: Phosphoenolpyruvate carboxykinase; PH: Pleckstrin homology; PI3K: Phosphatidylinositol-3 kinase; PIP2: Phosphatidylinositol (4,5)-bisphosphate; PIP3: Phosphatidylinositol (3,4,5)-triphosphate; PKB: Protein kinase B; PKCζ: Protein kinase C zeta; PPAR: Peroxisome proliferator activated receptor; PTEN: Phosphatase and tensin homolog deleted on chromosome 10; RNS: Reactive nitrogen species; ROS: Reactive oxygen species; S1P: Sphingosine-1-phosphate; SM: Sphingomyelin; SMase: Sphingomyelinase; SPT: Serine palmitoyl transferase; SREBP-1c: Sterol regulatory binding protein 1C; TLR4: Toll-like receptor 4; TNF-α: Tumor necrosis factor-alpha; TZD: Thiazolidinediones; UPR: Unfolded protein response; ZDF: Zucker diabetic fatty.

## Competing interests

The authors declare that they have no competing interests.

## Authors’ contributions

SG and AR drafted the first version of the manuscript. SP, AG and FT participated in researching the literature and critical discussion of the work. All authors read and approved the final manuscript.
